# Reconsidering LINE-1’s role in cancer: does LINE-1 function as a reporter detecting early cancer-associated epigenetic signatures?

**DOI:** 10.1093/emph/eoab004

**Published:** 2021-02-14

**Authors:** Maxfield M G Kelsey

**Affiliations:** Department of Molecular Biology, Cell Biology and Biochemistry, Brown University, Providence, RI 02912, USA

**Keywords:** LINE-1, cancer, epigenetics, metabolism

## Abstract

Long interspersed nuclear element-1 (LINE-1 or L1) is the only autonomously active retrotransposon in humans. While L1 has been implicated in several pathologies and the aging process, I present a model which challenges an understanding of L1 as predominantly antagonistic to human health. I hypothesize that L1 serves as a reporter in an early cancer alert system: a tripwire strung throughout the genome poised to trigger p53 and a type I interferon (IFN-1) response when the epigenetic landscape portends cancer. Cell proliferation and a shift to aerobic glycolysis cause dramatic changes in the epigenome which are permissive to L1’s escape from suppression. L1 has several properties which make it particularly apt to fulfill this hypothesized sentinel function. Being present in many copies spread throughout the genome allows it to monitor many regions for epigenetic instability and renders it robust to deactivation by mutation. This proposed cancer alert system would alter the cancer cell fitness landscape discouraging the use of growth-favoring aerobic glycolysis by threatening the activation of tumor-suppressive mechanisms. It also imposes costs on a strategy of non-specific global transcriptional derepression aimed at activating oncogenes. Erroneous activations of this system are predicted to increase the rate of aging, suggesting this represents a case of antagonistic pleiotropy trading prolonged youth for cancer prevention. More research is needed to assess this model.

**Lay summary:** During carcinogenesis the epigenome is remodeled by the Warburg effect and cellular proliferation. These processes globally relax chromatin. This epigenetic environment is permissive to the retrotransposon long interspersed nuclear element-1’s (LINE-1 or L1) escape from suppression. I hypothesize and present evidence for the notion that L1 has been co-opted to serve as a reporter in an early cancer alert system, poised to trigger tumor suppressive mechanisms when the epigenetic landscape portends cancer. This hypothesis describes a potentially major means by which transformation is thwarted early on.

## INTRODUCTION

The transposable elements (TEs) found in eukaryotic genomes have an evolutionary history dating back hundreds of millions of years [1]. They are ‘selfish’ genes, and some remain capable of spreading copies of themselves in the germline [1–3]. Long interspersed nuclear element-1 (LINE-1 or L1) is a non-long terminal repeat retrotransposon [2]. Copies of L1 account for a full 17% of the human genome [4]. Some of these copies are considered ‘active’ as they can replicate themselves utilizing a ‘copy and paste’ mechanism, inducing genetic mutations in doing so. As such, L1 is widely considered an injurious endogenous agent with carcinogenic potential [5–7]. The current understanding of L1 as predominantly antagonistic to human health would appear consistent with the fact that active L1 is detected in almost half of human cancers [8, 9]. L1 is the only autonomously active TE in humans and so it stands in stark contrast to all other transposons which have lost this ability [10]. Why might L1 defy this general trend? L1 is frequently envisioned as engaging in an ongoing arms race with the rest of our genome [11]. But within any conflict exists opportunity for cooperation [12]. It has been suggested that L1 insertion has been an important force in providing variation during human evolution and that it has a role in regulating early development [13, 14]. Could the L1/host relationship have other symbiotic characteristics?

## HYPOTHESIS

I suggest that the current understanding of L1’s relationship to cancer may in some ways be backwards. I hypothesize that L1 identifies and reports on signatures of cancerous epigenetic states to activate tumor suppression pathways.

## THE CANCER EPIGENOME

A dysregulated epigenome is a hallmark of cancer and is thought to occur in the earliest stages of tumorigenesis [15–20]. A global opening of chromatin involving changes in many epigenetic marks including DNA methylation and histone methylation, acetylation and lactylation has been well documented [15, 16, 21–23]. There exists much inter and intra-patient heterogeneity in the cancer epigenome, and its etiology is complicated [15–17]. Nevertheless, two epigenetic modulating processes are fundamental in carcinogenesis: sustained proliferation and an altered metabolism.

Global hypomethylation of the genome is associated with cell proliferation and cancer [24–29]. During periods of prolonged proliferation, cells have been observed to deplete their DNA of methylation, an epigenetic mark which generally acts to condense chromatin [16, 26, 30]. Global hypomethylation has also been shown to promote cellular capacity for self-renewal in certain stem cells [27, 28]. Whether altered DNA methylation induces or results from cell proliferation, the observation that cancer cells have been found to contain large hypomethylated blocks of DNA encompassing roughly half the genome is well established [24, 25, 29].

Another important driver of the shifting epigenetic landscape in cancer is the metabolic reprogramming induced by the Warburg effect [22]. This modified metabolism, termed aerobic glycolysis, is thought to afford cancer cells varied anabolic advantages such as increasing their pool of available biosynthetic precursors [31, 32]. As such, the metabolome is dramatically altered and this has epigenetic consequences [33–35]. The high lactate concentration observed during aerobic glycolysis has been found to stimulate gene expression by histone lactylation [21]. Aerobic glycolysis increases cellular butyrate concentration and butyrate can act as a histone deacetylase (HDAC) inhibitor in this context [36]. The increase in acetyl-coenzyme A concentration observed during the Warburg effect has been correlated with a global increase in histone acetylation, which acts to open chromatin [22, 23, 37, 38]. Accordingly, there are many metabolic mediators involved in the epigenetic modulation induced by the Warburg effect which act to globally decondense chromatin.

## L1 AS A REPORTER OF CANCEROUS EPIGENETIC SIGNATURES

It stands to reason that the dysregulated epigenetic environment observed during carcinogenesis is permissive to L1’s escape from suppression: tightly repressed by heterochromatin in healthy cells, L1 awakens to report on the oncogenic epigenetic reprogramming associated with cell proliferation and aerobic glycolysis [39]. Perhaps either the Warburg effect or proliferation is sufficient, given enough time, to remodel the epigenome and induce active L1 expression. Alternatively, perhaps both are necessary conditions for this to occur.

## L1 ACTIVATES DOWNSTREAM CANCER SUPPRESSION PATHWAYS

P53, a canonical tumor suppressor, is activated by L1 insertional DNA damage [40, 41]. It has been demonstrated that L1 activation can cause cancer cell growth arrest and apoptosis in a p53 dependent manner [41–43]. Also, L1 activation has been found to result in the accumulation of cytosolic L1 cDNA, which has been observed to trigger an interferon (IFN)-1 response [44]. L1 activation can both induce cellular senescence and maintain the senescence-associated secretory phenotype (SASP) [45–47]. The induction of an interferon response and the SASP encourages clearance by the immune system [48, 49]. L1 can therefore activate p53, cellular senescence and immune clearance pathways, established cancer suppression mechanisms [49–51]. The proposed model furnishes these mechanisms with a cancer sensing trigger.

## ADAPTIVE L1 FEATURES RELATIVE TO ITS PROPOSED ROLE

But why should L1, quite the labile genetic element, be retained to serve this function as opposed to a potentially less dangerous gene? Two of L1’s structural features make it particularly apt for this role. That L1 elements are spread throughout the genome makes it well suited to act as an epigenetic sentinel: many different regions can be monitored for instability. That L1 is present in many copies renders it particularly robust against simple deactivation by loss-of-function mutation. Furthermore, an evolutionary point of view reveals reasons why co-opting L1 for such a capacity may have been readily feasible. Having taken part in an arms race with the rest of the genome, the machinery to detect and suppress L1 would have already been present—L1 had merely to be tamed and the machinery repurposed. There is evidence that p53 has had such a relationship with L1: not only is it activated by L1, it also seems to act as an L1 transcriptional repressor [52]. Another Darwinian insight lies in recognizing how this putative system might constrain cancer evolution at a cellular level. L1 signaling would alter the cancer cell fitness landscape. It discourages the otherwise beneficial use of growth-favoring aerobic glycolysis by threatening the activation of p53 and an IFN-1 response. Similarly, it imposes costs on a strategy of non-specific global transcriptional derepression aimed at activating oncogenes.

## TUNING THE L1 REPORTER SYSTEM

A signal detection framework applies to determine the optimal tuning of this reporter system: too hair-trigger a system will mark benign cells for clearance and needlessly inflame tissues. Too high an action threshold and some incipient cancers will fail to be detected. An obvious and crude potential tuning mechanism is modulation of active L1 copy number. The greater the number of activatable elements, the easier it will be to marshal a signaling equivalent of L1; in other words, the greater the sensitivity and the lower the specificity. There is evidence supporting the notion that selection has tuned L1 copy number in humans. Our genome contains more than 500 000 L1 sequences, but almost all of these have been inactivated by mutation [2]. Only around 80–100 copies are considered active [53–55]. This fact also suggests that these retained active copies are adaptive: were they merely deleterious, it would be quite strange if selection was able to disable 499 900 copies, but could not manage this for the remaining 100. Another more delicate tuning mechanism might be in the selection of active L1 elements by promoter characteristics. It has been reported that cancer-induced global hypomethylation differentially influences the methylation status of L1 loci [56]. L1 elements whose promoter regions show the most sensitive and specific derepression in response to cancer’s permissive effects on chromatin should be those which are retained in active form.

## TRADEOFFS

As depicted, this system comes at a cost. In helping protect from cancer, the inevitable erroneous activations of the system will cause benign tissue to be damaged by sterile inflammation and inappropriate p53 activation will result in a loss of cellularity and a faster depletion of stem cell replicative potential. Both of these effects have been implicated in the aging process [57]. L1 functioning as hypothesized therefore constitutes a case of antagonistic pleiotropy between cancer and aging. This is an interesting dynamic, seeing as cancer is itself a disease of aging. While it is likely that the damage caused by erroneous L1 activation can increase the risk of cancer, activable L1 should nevertheless not increase net cancer likelihood given its role in mediating tumor suppression. Evidence for the pro-aging portion of this pleiotropy has been observed. It is established that L1 derepression during aging can drive tissue inflammation, speeding up the aging process [44, 47]. Furthermore, knocking out SIRT6, a histone deacetylase which helps repress L1 transcription, leads to a progeroid phenotype marked by a decreased mean and maximum lifespan in mice [47].

## MODEL PREDICTIONS

While this accumulating body of evidence is suggestive, it by no means establishes this model as correct. This model is readily testable as it makes many falsifiable predictions. The most fundamental predictions are that if you supernormally repress or delete L1 you should get earlier cancer occurrence and a slower rate of aging. Conversely, were you to have abnormally low L1 repression, later cancer occurrence and more rapid aging are predicted. Another prediction, described in a previous section but restated here, is that L1 elements whose promoter regions show the most sensitive and specific derepression in response to cancer’s permissive effects on chromatin should be those which are retained in active form.

## CLINICAL OBSERVATIONS

According to this model, the roughly 50% of cancers we observe clinically which are L1 positive must be those that have managed to disarm key elements in this suppression strategy. This is supported by the finding that nearly all L1 positive cancers have mutated p53 [9]. Note that with p53 or other components of this cancer prevention system mutated such that L1 signaling does not halt growth, L1 activation might become a clinical detriment by permitting faster cancer evolution through L1 mediated insertional mutagenesis. Some data support this notion, such as the finding that a higher degree of L1 hypomethylation in colorectal cancer is associated with more advanced disease [58] and that L1 insertional mutagenesis of tumor suppressor genes has been observed [59]. In contrast, cancers which are L1 negative will have managed to avoid this detection system, perhaps through maintaining better control over their epigenome by adhering to a more normal metabolic state.

Other clinical observations lend support to this model. Many cancers have been found to overexpress factors which condense chromatin—this would constitute a logical means stifling L1 expression. For instance, Li and Seto report that many cancers overexpress HDACs [60]. Furthermore, SETDB1, a DNA methyl transferase which is known to silence TE expression, is overexpressed in many cancers [61]. These observations are consistent with the notion that successful cancer cells must work to silence L1 in the face of the global transcriptional derepression accompanying proliferation and aerobic glycolysis. Nevertheless, these enzymes have broad epigenetic effects and are involved in many cellular processes; their overexpression is by no means necessarily related to L1 inactivation.

Assessing the overall impact this system has on cancer suppression is difficult from clinical data, which tends to capture disease that has progressed well beyond early transformation. It would be informative to know the fraction of L1 positive, transformed cells which self-abort early in cancer progression. Live imaging of cells containing both an oncogene and a fluorescent reporter of active L1 could go some way toward answering this important question.

## CONCLUDING REMARKS

This hypothesis describes a potentially major means by which transformation is thwarted early on. If accurate, it has significant implications in oncology and gerontology. L1 negative cancers could possibly be responsive to treatments activating L1 using epigenetic modulators, such as DNA demethylases or histone acetyltransferases. While the cancer/aging tradeoff highlighted in this model casts doubts on the suppression of L1 as a viable longevity enhancing strategy for a general population, such an approach may hold promise toward ameliorating particular pathologies associated with L1. Taken together, the data reported on in this article are consistent with the model presented but more research is needed to better assess its veracity. To this end, several model predictions have been outlined.

**Conflict of interest:** None declared.
